# Dementia Diagnosis Is Associated with Changes in Antidiabetic Drug Prescription: An Open-Cohort Study of ∼130,000 Swedish Subjects over 14 Years

**DOI:** 10.3233/JAD-200618

**Published:** 2020-08-18

**Authors:** Juraj Secnik, Hong Xu, Emilia Schwertner, Niklas Hammar, Michael Alvarsson, Bengt Winblad, Maria Eriksdotter, Sara Garcia-Ptacek, Dorota Religa

**Affiliations:** a Department of Neurobiology, Center for Alzheimer Research, Division of Clinical Geriatrics, Care Sciences and Society, Karolinska Institutet, Huddinge, Sweden; b Department of Medical Epidemiology and Biostatistics, Karolinska Institutet, Stockholm, Sweden; c Institute of Environmental Medicine, Karolinska Institutet, Stockholm, Sweden; d Department of Molecular Medicine and Surgery, Growth and Metabolism, Karolinska Institutet, Stockholm, Sweden; e Department of Neurobiology, Center for Alzheimer Research, Division of Neurogeriatrics, Care Sciences and Society, Karolinska Institutet, Stockholm, Sweden; f Theme Aging, Karolinska University Hospital, Huddinge, Sweden; g Department of Internal Medicine, Södersjukhuset, Section for Neurology, Stockholm, Sweden

**Keywords:** Dementia, diabetes mellitus, hypoglycemic agents, pharmacoepidemiology

## Abstract

**Background:**

Care individualization dominates in clinical guidelines for cognitively impaired patients with diabetes; however, few studies examined such adaptations.

**Objective:**

Describe long-term pharmacological changes in diabetes treatment in subjects with and without dementia.

**Methods:**

We performed a registry-based cohort study on 133,318 Swedish subjects (12,284 [9.2%] with dementia) with type 2 or other/unspecified diabetes. Dementia status originated from the Swedish Dementia Registry, while the National Patient Register, Prescribed Drug Register, and Cause of Death Register provided data on diabetes, comorbidities, drug dispensation, and mortality. Drug dispensation interval comprised years between 2005 and 2018 and the dispensation was assessed relative to index date (dementia diagnosis) in full cohort and propensity-score (PS) matched cohorts. Annual changes of drug dispensation were analyzed by linear regression, while Cox and competing-risk regression were used to determine the probability of drug dispensation after index date in naïve subjects. Studied medications included insulin, metformin, sulfonylureas, thiazolidinediones, dipeptidyl-peptidase-4 inhibitors (DPP-4i), glucagon-like peptide-1 agonists (GLP-1a), and sodium-glucose cotransporter-2 inhibitors (SGLT-2i).

**Results:**

Dementia patients had higher probability of insulin dispensation (hazard ratio 1.21 [95% CI 1.11–1.31] and lower probability of DPP-4i (0.72 [0.66–0.79]), GLP-1a (0.51 [0.41–0.63]), and SGLT-2i dispensation (0.44 [0.36–0.54]) after index date. PS-matched analyses showed increased annual insulin dispensation (β difference 0.97%) and lower increase in DPP-4i (–0.58%), GLP-1a (–0.13%), and SGLT-2i (–0.21%) dispensation in dementia patients compared to dementia-free controls.

**Conclusion:**

Dementia patients had lower probability of receiving newer antidiabetic drugs, with simultaneous higher insulin dispensation compared to dementia-free subjects.

## INTRODUCTION

The clinical co-occurrence of diabetes mellitus (diabetes) and dementia is becoming increasingly more common as the care for chronic disorders improves and life-expectancy rises. It is currently estimated that approximately every 6th patient diagnosed with dementia is also living with diabetes [1, 2].

Relationship between dementia and diabetes is complex, as diabetes contributes to cognitive decline [3] and good neurocognitive performance is an essential prerequisite for successful self-management of diabetes [4]. However, it is not clear how diabetes management should be adapted when dementia is present. Avoiding hypoglycemia is one of the main therapeutic goals, as cognitive impairment increases hypoglycemia risk [5] and severe hypoglycemia predisposes patients to worse cognitive performance [6], in a vicious circle. On the other hand, increases in HbA1c levels are significantly associated with worse cognitive functioning [7]; however, evidence points against stringent adherence to glycemic targets as it does not seem to provide cognitive benefit in advanced ages [8]. As a consequence, regime simplification, individualization, and relaxed glycemic targets dominate in the current guidelines for diabetes management in the cognitively impaired older patients [4, 9, 10].

Importantly, the current spectrum of pharmacological treatments of diabetes (type 2 diabetes specifically) does allow for significant adaptations based on the needs of individual patients. Metformin is still the primary option for most, with advanced kidney disease and liver failure posing the main prescription caveats. In addition, metformin’s neurocognitive effects have not yet been determined [11, 12].

Among sulfonylureas, shorter-acting drugs should be preferred due to higher propensity to hypoglycemia [4], and possibly worse cognitive outcomes in comparison to metformin [13]. Thiazolidinedione (TZD) prescription should be closely monitored due to known risk of congestive heart failure and fractures [14].

Within incretin-based therapies, dipeptidyl-peptidase-4 inhibitors (DPP-4i) provide the easier regimen, as injections of glucagon-like peptide-1 agonists (GLP-1a) require unimpaired visuo-motor coordination [15]. The CARMELINA trial failed to show cognitive improvements in DPP-4i users [16]; however, GLP-1a animal models promise modulation of both amyloid and tau pathologies and possibly dual Alzheimer’s-diabetes treatment [17]. Sodium-glucose cotransporter-2 inhibitors (SGLT-2i) exhibit multiple benefits additional to glycemic control [18]; on the other hand, volume depletion might be a clinical concern in the older patients [4].

Lastly, insulin carries the highest hypoglycemic risk; however, if adjustments are made (e.g., once-daily basal insulin with morning application) and assistance is provided, insulin can be a sensible choice in older patients with type 2 diabetes [4].

However, it is unclear how the management of diabetes is adjusted in routine clinical practice in the lead-up to dementia diagnosis and with advancing cognitive impairment and loss of independence.

The aim of this study was to describe the differences in pharmacological care of diabetes in the interval prior to and after diagnosis of dementia in a large Swedish registry-based cohort. Secondly, we wanted to investigate how the overall prescription of specific antidiabetic medication differs in patients with and without dementia within a wide time interval.

## MATERIAL AND METHODS

This prospective-cohort study was based on merged data from five Swedish national registers and one longitudinal database with the unique personal identification number (personnummer, mandatory for Swedish citizens) used to link data across sources. The National Board of Health and Welfare and Statistics Sweden co-participated on the data merge and anonymization of data. We describe the study population, and the data sources used with corresponding variables originating from the respective registers.

### Study population: Summary

The original data extraction comprised 1,752,659 subjects: 424,624 (24.2%) patients diagnosed with dementia and up to four matched dementia-free controls (1,328,035 [75.8%]; see section on the Swedish Total Population Register [Population Register]). Out of this population, we selected only the sub-population of subjects with diabetes and restricted the diagnosis of dementia to originate only from the Swedish Dementia Registry (SveDem, 184,560 subjects). After applying further exclusion criteria, 133,318 subjects with diabetes mellitus (12,284 [9.2%] with dementia) were analyzed using the whole-cohort analysis. In addition, propensity-score matching was performed to compare only patients with similar characteristics (see Supplementary Figure 1).

### Swedish Dementia Registry and Swedish Total Population Register

SveDem is a Swedish quality-of-care registry for dementia and has been thoroughly described previously [19]. SveDem was established in 2007 with the purpose to register all dementia patients in Sweden at the time of diagnosis and standardize their care. Patient variables include clinical characteristics (e.g., Mini-Mental State Examination [MMSE]), sociodemography (e.g., living arrangements), community support (e.g., daycare), and chronic pharmacological treatment [19]. Dementia disorders recorded in SveDem include Alzheimer’s disease, mixed-pathology dementia, vascular dementia, dementia with Lewy bodies, frontotemporal dementia, Parkinson’s disease dementia, unspecified dementia and other dementia types. In 2018, more than 82,000 dementia patients have been registered, which makes SveDem the largest dementia registry in the world [20].

Population Register is available in computerized form since 1968 and is the basis for official population statistics for large parts of Statistics Sweden’s operations [21]. This register provided dementia-free controls for the patients with dementia.

#### Dementia cases

To improve precision of dementia cases, only diagnoses of dementia from SveDem were included, while patients with dementia diagnoses included in other registers (see exclusion criteria for controls below) and not recorded by SveDem were excluded. Specifically, only patients diagnosed with any dementia and registered to SveDem between May 1, 2007 until October 16, 2018 were included (80,004 patients [18.8% out of all dementia patients in the original data - 424,624 patients]).

#### Dementia-free controls

In the original data extraction, a pool of dementia-free subjects was extracted from the Population Register. The exclusion criteria for controls were as follows: a) having dementia diagnosis recorded in SveDem; b) or ICD-10 codes F00-F03, G30, G31 (see Supplementary List 1) recorded by the Swedish National Patient Register (Patient Register) or Swedish Cause of Death Register (Death Register); c) or having ATC code N06D (anti-dementia drugs) recorded by the Swedish Prescribed Drug Register (Drug Register); To improve the pool of eligible controls, subjects who had ICD-10 codes F05-F09, G32 (Supplementary List 1) were not considered as controls (nor as dementia cases). Consequently, up to four dementia-free controls per one dementia case were matched with dementia cases on birth year (±3 years), sex, and the county of residence and assigned an index date matching with the dementia diagnosis date (1,328,035 controls matched with 424,624 cases in total).

Afterwards, only subjects diagnosed with diabetes with and without diagnosis of dementia, where dementia diagnosis originated only from SveDem were selected (184,560 subjects in total). After excluding subjects with incorrect or missing data and patients with type 1 diabetes, the cohort consisted of 133,318 subjects with diabetes, while 12,284 (9.2%) had diagnosis of dementia and 121,034 (90.8%) were dementia-free (Supplementary Figure 1). This dementia –dementia-free population with diabetes was the basis for the whole-cohort analysis. In addition, 1:1 propensity score (PS) matching was used to create a pool of comparable dementia –dementia-free pairs with diabetes. As a convention, the term “index date” will refer to both date of dementia diagnosis in the dementia cohort and assigned index date in the dementia-free cohort. Detailed description of the dementia cohort is summarized in Supplementary Table 1.

### Swedish National Patient Register

The Patient Register [22] contributed records on inpatient diagnoses since 1998 and specialized outpatient diagnoses since 2001 in Sweden. During the studied time interval, the included diagnoses were coded according to the 10th version of the International Classification of Diseases (ICD-10) [23]. Data were extracted until December 31, 2017.

#### Diabetes mellitus

Diabetes was identified by the ICD-10 codes E10–E14 in the Patient Register or by antidiabetic treatment (ATC code A10) included in the Swedish Prescribed Drug Register (Drug Register) prior to and including the index date. Subsequently, diabetes was grouped into three types: type 1 diabetes, type 2 diabetes, and other/unspecified diabetes (for details on extraction and coding, see Supplementary Algorithm 1). Duration of diabetes was based on the difference between the index date and either the date of the earliest record in the Patient Register where diagnosis of diabetes occurred, or the earliest dispensation date of ATC code A10 from the Drug Register. Only patients with type 2 diabetes and other/unspecified types were considered in the analyses, as inclusion of type 1 diabetes would increase the proportion of patients receiving insulin.

#### Comorbidities

To adjust for the effect of additional chronic diseases, we created a comorbidity index as described by Charlson et al. [24], using the algorithm described by Quan et al. [25] as a weighted sum of diagnosed chronic disorders up to and including index date. The codes referring to the renal diseases were not included in the index but extracted as a separate adjustment/matching variable because renal disease has significant overall effect on antidiabetic drug prescription. Diabetes variables were omitted from the index to avoid over-adjustment and the index was increased by one point for dementia patients.

### Longitudinal integrated database for health insurance and labor market studies (LISA)

LISA is an administrative database and provides accurate statistics in health and labor market research [26]. Data in LISA covers the adult Swedish population since 1990, and includes information on sick leave, disability pensions, education, income, and other socioeconomic characteristics, with high level of information completeness [26].

#### Education

The highest attained education for every patient at the time of index date was extracted from LISA and grouped into seven categories from the lowest (<9 years of completed education) to the highest attained education (doctoral/research education).

#### Disposable income

Disposable income in Swedish Krona (SEK) at the time of index date inflated on the 2019 value of Consumer Price Index was extracted from LISA, and grouped into three categories, with 33rd and 66th percentiles used as cut-offs to create categories (low, middle, high income).

### Swedish Prescribed Drug Register

The Drug Register, established in 2005, includes data on all dispensed drug prescriptions at Swedish pharmacies [27]. The pharmacological records are coded according to the Anatomical Therapeutic and Chemical (ATC) classification. Drug dispensations included in this study occurred between the start of the register until December 31, 2018.

#### Diabetes mellitus

ATC codes A10 (drugs used in diabetes), A10A (insulins), and A10B (blood glucose lowering drugs excluding insulin) before and after the index date extracted from the Drug Register were used in combination with the Patient Register to identify overall diabetes prevalence and classify diabetes types (see Supplementary Material 1).

#### Antidiabetic drug dispensation

Seven antidiabetic drug classes were extracted from the Drug Register according to following ATC codes: insulin (A10A), metformin (A10BA02), sulfonylurea derivates (SU) (A10BB), thiazolidinediones (TZD) (A10BG), DPP-4i (A10BH), GLP-1a (A10BJ), and SGLT-2i (A10BK). Dispensations of the individual medication classes were extracted on a yearly basis relative to the index date (dementia diagnosis date in the dementia cohort) (e.g., dispensation of A10A in the one-year period prior to index date). As subjects’ index date spanned from 2007 to 2018, the range of dispensation data allowed for extraction of fourteen years of antidiabetic drug dispensation prior to the latest index date and twelve years after the earliest index date. For example, a subject with index date on January 1, 2012 could have contributed seven years of possible drug dispensation data before and seven years after index date (provided the patient survived until the end of study –December 31, 2018). A subject was considered as user of medication if a dispensation was recorded at least once in the one-year period relative to the index date. For the time-to-drug dispensation analysis, we also extracted the first dispensation date of individual medication classes after the index date.

#### Supplementary medication

In the multivariate and matched analyses, we also used the dispensation of cardiovascular (ATC code C), antithrombotic (B01), antipsychotic (N05A), antidepressant (N06A), anxiolytic (N05B), and hypnotic/sedative drugs (N05C) up to three years prior to and including the index date as recorded by the Drug Register.

### Swedish Cause of Death Register (Death Register)

The Death Registry contains data starting 1952 and is the basis for official statistics on death causes in Sweden [28]. The purpose of the registry is to describe the development of national all-cause and specific-cause mortality.

#### Mortality

We extracted the information from the Death Registry since its initiation until December 31, 2018, the end of the study follow-up. Overall mortality was considered if a valid record (patient death dated after dementia diagnosis) was present.

#### Statistical analysis

The presence of dementia was the exposure of interest, and the whole-cohort and PS-matched approaches to analyses were used. The whole cohort consisted of 133,318 subjects. Second, for the descriptive and univariate analyses, 1:1 PS nearest-neighbor matching with 0.1 caliper of the logit of the propensity score combined with exact matching on the index year (year of the index date) was used to create the dementia–dementia-free pairs. Characteristics used to generate PS included age, sex, Charlson comorbidity score, renal disease, diabetes type, diabetes duration, attained education, income category, and use of cardiovascular, antithrombotic, antipsychotic, antidepressant, hypnotic/sedative, and anxiolytic drugs. In total, 11,938 dementia –dementia-free PS-matched pairs were identified for the descriptive and univariate analyses (97.2% of the original dementia cohort was retained).

In the time-to-drug dispensation approach (survival analysis), probability of dispensation after index date was assessed in persons without history of the specific drug dispensation prior to and including the index date (naïve users). In the whole cohort, a restriction was made in the regression modelling, while in the PS analyses only subjects without prior drug history were used for matching. Three populations were analyzed: a) all naïve users; b) naïve users who were using metformin in the one-year period prior to and including index date (add-on therapy); c) all naïve users within strata of index years. PS-matching was done similarly as in univariate analyses, with the addition of matching on other antidiabetic medication dispensed prior to or including the index date in all three analyses, while exact matching on index year was omitted in the year-stratified analysis. For the analysis of all subjects, 7,284 pairs were generated for insulin dispensation, 3,578 for metformin, 7,892 for sulfonylurea, 11,506 for TZD, 11,081 for DPP-4i, 11,771 for GLP-1a and 11,852 for SGLT-2i (see Supplementary Tables 2–8). For the analyses of patients who were using metformin, 4,067 pairs were generated for insulin dispensation, 3,964 for sulfonylurea, 5,852 for TZD, 5,603 for DPP-4i, 6,002 for GLP-1a, and 6,030 pairs for SGLT-2i dispensation. The number of PS-matched pairs in the year-stratified analyses are summarized in Supplementary Table 9.

#### Descriptive and univariate analyses

Differences in baseline characteristics between the dementia and dementia-free cohorts were assessed using chi-square, independent samples *t*-test, and ANOVA, and their non-parametric equivalents. Standardized mean differences (SMDs) were used to assess balance in the propensity-score matched cohorts.

Annual percentages of seven specific antidiabetic medication groups were estimated as a proportion of patients dispensed a specific drug out of all patients dispensed any antidiabetic drug in the respective year (e.g., proportion of insulin users that year out of all antidiabetic drug users that year). Linear regression was used to model percentual annual change in antidiabetic drug proportions. These proportions and yearly changes in specific anti-diabetic drug classes were assessed in both the PS-matched and the whole cohort.

#### Survival analyses

Crude and adjusted hazard ratios (HR) and subdistribution hazard ratios (sHR) were determined using Cox proportional hazard regression and competing risk regression models according to Fine and Gray, respectively, with death as competing event in Fine and Gray models. Time to first dispensation of medication after index date in patients without prior medication history were the primary events of interest. Attained age was used as timescale in the analyses of all naïve users and in the metformin-add on analyses, while time-since entry was used in the year-stratified analysis. Proportionality of hazards assumptions were examined with modelling Schoenfeld residuals as a function of time and testing the hypothesis of zero-slope. If non-proportionality was detected, variable-time interactions were introduced. Statistical significance was determined using *p*-values and 95% confidence intervals (CI). Missing information were excluded prior to analysis (see Supplementary Figure 1).

Data were analyzed using Stata v16 (Stata Statistical Software: Release 16. StataCorp LLC, College Station, TX) and SPSS version 23 (IBM Corp., Armonk, NY).

### Ethical considerations

Data are covered by a specific ethical approval and the extraction, linkage, and anonymization were performed by two government agencies, the National Board of Health and Welfare and Statistics Sweden. *Researches were provided only with anonymized data and no link could be made to an individual. Study complies with the Declaration of Helsinki and was approved by the regional ethical committee in Stockholm, Sweden (number of the ethical approval: 2017/501-31).

## RESULTS

After applying the study selection criteria, the final cohort consisted of 133,318 patients with type 2 diabetes or other/unspecified diabetes. Overall, 12,284 (9.2%) subjects had a diagnosis of dementia and 121,034 were dementia-free. The PS-matched cohort used for univariate and descriptive statistics consisted of 11,938 pairs (Supplementary Figure 1).

### Univariate analyses

Univariate results are summarized in Tables 1 and 2 and Supplementary Figures 2 and 3. In the full cohort, dementia patients were younger (79.7 versus 80.6 years), had longer diabetes duration (7.5 versus 6.5 years), higher comorbidity burden (2 versus 1), and significantly higher dispensation of multiple psychotropic drugs.

**Table 1:** Baseline characteristics in the dementia and dementia-free cohort

	Diabetes - Whole cohort			Diabetes - PS-matched cohort				
Dementia 12,284 (9.2%)	Dementia-free 121,034 (90.8%)	*p*	Dementia 11,938 (50.0%)	Dementia-free 11,938 (50.0%)	*p*	SMD
Age, y	79.7 (7.1)	80.6 (7.2)	<0.001	79.7 (7.2)	79.6 (7.4)	0.32	0.02
Female	6,302 (51.3%)	59,861 (49.5%)	<0.001	6,082 (50.9%)	5,856 (49.1%)	0.29	0.02
Diabetes Type							
Type 2	7,751 (63.1%)	71,558 (59.1%)	<0.001	7,532 (63.1%)	7,827 (65.6%)	<0.001	0.04
Other/Unspecified	4,533 (36.9%)	49,476 (40.9%)	4,406 (36.9%)	4,111 (34.4%)
Diabetes duration, years	7.5 (6.4)	6.5 (6.4)	<0.001	7.5 (6.4)	7.9 (6.5)	<0.001	–0.09
Charlson comorbidity index	2 (2)	1 (2)	<0.001	2 (2)	2 (2)	<0.001	0.05
Renal disease	825 (6.7%)	9,006 (7.4%)	0.003	807 (6.8%)	844 (7.1%)	0.35	–0.01
Cardiovascular drugs	11,707 (95.3%)	115,837 (95.7%)	0.036	11,387 (95.4%)	11,397 (95.5%)	0.76	–0.01
Antithrombotic drugs	9,148 (74.5%)	86,277 (71.3%)	<0.001	8,911 (74.6%)	8,970 (75.1%)	0.38	–0.02
Antipsychotics	930 (7.6%)	3,792 (3.1%)	<0.001	878 (7.4%)	731 (6.1%)	<0.001	0.07
Antidepressants	4,546 (37.0%)	24,985 (20.6%)	<0.001	4,396 (36.8%)	4,491 (37.6%)	0.20	–0.01
Hypnotics/Sedatives	4,218 (34.3%)	38,570 (31.9%)	<0.001	4,082 (34.2%)	4,044 (33.9%)	0.60	0.02
Anxiolytics	2,917 (23.7%)	23,538 (19.4%)	<0.001	2,808 (23.5%)	2,815 (23.6%)	0.92	–0.01
Education						
<9 years compulsory	5,258 (43.9%)	56,044 (47.6%)	<0.001	5,247 (44.0%)	5,138 (43.0%)	0.16	–0.05
9 years compulsory	896 (7.5%)	7,434 (6.3%)	892 (7.5%)	817 (6.8%)
2 years upper secondary	3,092 (25.8%)	29,305 (24.9%)	3,083 (25.8%)	3,147 (26.4%)
3 years upper secondary	1,081 (9.0%)	9,996 (8.5%)	1,074 (9.0%)	1,132 (9.5%)
<3 years college	688 (5.7%)	6,596 (5.6%)	684 (5.7%)	730 (6.1%)
3 years college	887 (7.4%)	7,624 (6.5%)	879 (7.4%)	905 (7.6%)
Research education	80 (0.7%)	621 (0.5%)	79 (0.7%)	69 (0.6%)
Income category							
Low	3,737 (30.4%)	40,175 (33.2%)	<0.001	3,541 (29.7%)	3,378 (28.3%)	0.002	–0.04
Middle	4,222 (34.4%)	39,787 (32.9%)	4,118 (34.5%)	4,028 (33.7%)
High	4,317 (35.2%)	41,040 (33.9%)	4,279 (35.8%)	4,532 (38.0%)
Overall antidiabetic drug use							
Insulin	6,271 (51.1%)	57,867 (47.8%)	<0.001	6,086 (51.0%)	6,016 (50.4%)	0.37
Metformin	9,023 (73.5%)	84,107 (69.5%)	<0.001	8,756 (73.3%)	8,559 (71.7%)	<0.001
Sulfonylureas	4,452 (36.2%)	43,835 (36.2%)	0.96	4,315 (36.1%)	4,149 (34.8%)	0.025
TZD	461 (3.8%)	4,344 (3.6%)	0.35	453 (3.8%)	449 (3.8%)	0.89
DPP-4i	1,689 (13.7%)	17,220 (14.2%)	0.15	1,652 (13.8%)	1,911 (16.0%)	<0.001
GLP-1a	313 (2.5%)	3,637 (3.0%)	0.004	303 (2.5%)	483 (4.0%)	<0.001
SGLT-2i	244 (2.0%)	3,580 (3.0%)	<0.001	239 (2.0%)	477 (4.0%)	<0.001
Mortality	6,514 (53.0%)	56,389 (46.6%)	<0.001	6,320 (52.9%)	5,245 (43.9%)	<0.001

PS, propensity-score; SMD, standardized mean differences in PS-matched dementia cases vs controls; TZD, thiazolidinediones; DPP-4i, dipeptidyl-peptidase-4 inhibitors; GLP-1a, glucagon-like peptide-1 agonists; SGLT-2i, sodium-glucose co-transporter-2 inhibitors; Age is described as mean (SD); Diabetes duration and Charlson comorbidity index are described as median (IQR); Other variables are described as number of patients (%); Age, diabetes duration, Charlson comorbidity index, renal failure, education and income category are described at the time of index date; Subjects were considered users of antidiabetic medication if they had dispensation of the specific drug at any timepoint, before or after index date. Other medication use was determined in the interval index date and three-years prior; SMDs - calculated for matching variables.

**Table 2:** Proportion change in specific antidiabetic drug usage with annual increments

	Diabetes - Whole cohort		
	Dementia Cases β (95% CI)	Dementia-free Controls β (95% CI)	Absolute difference
Insulin	1.99% (1.65–2.34)^*^	1.37% (1.27–1.47)^*^	0.62% (D↑)
Metformin	–1.33% (–1.65;–1.00)^*^	–1.09% (–1.23;–0.94)^*^	0.24% (D↓)
Sulfonylureas	–1.38% (–1.09;–0.96)^*^	–1.38% (–1.58;–1.18)^*^	0%
TZD	–0.20% (–0.23;–0.17)^*^	–0.18% (–0.21;–0.14)^*^	0.02% (D↓)
DPP-4i	0.53% (0.47–0.60)^*^	0.84% (0.76–0.93)^*^	0.31% (ND↑)
GLP-1a	0.12% (0.07–0.17)^*^	0.19% (0.17–0.22)^*^	0.07% (ND↑)
SGLT-2i	0.06% (0.04–0.08)^*^	0.17% (0.14–0.20)^*^	0.11% (ND↑)
	Diabetes –PS-matched cohort		
	Dementia Cases β (95% CI)	Dementia-free Controls β (95% CI)	Absolute difference
Insulin	1.96% (1.61–2.31)^*^	0.99% (0.82–1.15)^*^	0.97% (D↑)
Metformin	–1.33% (–1.64;–1.02)^*^	–1.06% (–1.23;–0.89)^*^	0.27% (D↓)
Sulfonylureas	–1.34% (–1.55;–1.13)^*^	–1.04% (–1.24; –0.84)^*^	0.30% (D↓)
TZD	–0.21% (–0.24;–0.17)^*^	–0.22% (–0.25;–0.18)^*^	0.01 (ND↓)
DPP-4i	0.56% (0.49; 0.64)^*^	1.14% (1.00–1.29)^*^	0.58% (ND↑)
GLP-1a	0.13% (0.07–0.19)^*^	0.26% (0.17–0.35)^*^	0.13% (ND↑)
SGLT-2i	0.07% (0.01–0.12)^*^	0.28% (0.13–0.43)^*^	0.21% (ND↑)

TZD, thiazolidinediones; DPP-4i, dipeptidyl-peptidase-4 inhibitors; GLP-1a, glucagon-like peptide-1 agonists; SGLT-2i, sodium-glucose co-transporter-2 inhibitors; PS, propensity score; Beta coefficients represent slope of percent change in antidiabetic drug usage with advancing time (one-year increments) derived from the linear regression analysis (with one predictor); time period included 14 years prior and 12 years after index date (date of dementia diagnosis); D↑ larger percentual dispensation increase in the dementia cohort; D↓ larger percentual dispensation decrease in the dementia cohort; ND↑ larger percentual dispensation increase in dementia-free cohort; ND↓ larger percentual dispensation decrease in dementia-free cohort; ^*^p < 0.001.

After PS-matching, the differences between dementia and dementia-free subjects were substantially reduced and all standardized mean differences were below 0.1 SD. Covariate balances are summarized in Table 1 and Supplementary Tables 2–8 (balances in the PS-matched analyses of metformin users and specific index years are not shown). In the PS-matched cohorts, dementia patients experienced higher total utilization of metformin (73.3% versus 71.7%) and sulfonylureas (36.1% versus 34.8%) in comparison to dementia-free controls. Conversely, DPP-4i (13.8% versus 16.0%), GLP-1a (2.5% versus 4.0%), and SGLT-2i (2.0% versus 4.0%) were less commonly prescribed in dementia patients (Table 1).

The estimates from the linear regression in the matched cohort showed steeper yearly increase in insulin prescription in dementia patients compared to dementia-free controls (1.96% versus 0.99%; Table 2, Supplementary Figure 3). Both the dementia and dementia-free cohort experienced significant metformin and sulfonylurea de-prescribing with each year of follow-up (–1.33% versus –1.06% and –1.34% versus –1.04%, respectively). Simultaneously, DPP-4i (0.56% versus1.14%), GLP-1a (0.13% versus 0.26%), and SGLT-2i (0.07% versus 0.28%) dispensation increased; however, this increase was less pronounced in dementia patients. Trend in TZD prescribing was comparable in both exposure groups (–0.21% versus –0.22%). The whole-cohort analyses followed a similar pattern (Table 2, Supplementary Figure 2).

### Multivariate analyses

The time-to-drug dispensation analyses using Cox and competing risk regression are summarized in Tables 3 and 4. All analyses were performed in subjects without prior history of use of the respective antidiabetic drug, and separate analyses were performed in subjects who were users of metformin in the one-year period prior to or including index date (Table 4) and an analysis of subjects without prior medication history stratified on index year (Supplementary Figure 4).

**Table 3:** Probability of antidiabetic drug dispensation after index date in naïve patients

	Dementia versus Dementia-free (Whole-cohort analysis)	
	Cox regression Adjusted HR (95% CI)	Competing risk regression Adjusted sHR (95% CI)
Insulin (13,755 new dispensations)	1.28 (1.21–1.35)^ †^	1.22 (1.15–1.29)^ †^
Metformin (4,734)	1.03 (0.93–1.15)	1.02 (0.91–1.14)
Sulfonylureas (3,832)	0.93 (0.82–1.05)	0.90 (0.80–1.03)
TZD (335)	0.64 (0.40–1.03)	0.62 (0.39–1.00)
DPP-4i (9,802)	0.88 (0.81–0.95)^*^	0.84 (0.78–0.90)^ †^
GLP-1a (2,096)	0.59 (0.49–0.71)^ †^	0.56 (0.47–0.67)^ †^
SGLT-2i (2,794)	0.54 (0.45–0.64)^ †^	0.50 (0.43–0.60)^ †^
	Dementia versus Dementia-free (PS-matched analysis)	
	Cox regression Crude HR (95% CI)	Competing risk regression Crude sHR (95% CI)
Insulin (2,543 new dispensations)	1.29 (1.19–1.40)†	1.21 (1.11–1.31)†
Metformin (829)	0.90 (0.79–1.03)	0.88 (0.77–1.02)
Sulfonylureas (560)	0.95 (0.80–1.12)	0.90 (0.76–1.07)
TZD (45)	0.72 (0.40–1.30)	0.68 (0.38–1.23)
DPP-4i (1,789)	0.75 (0.69–0.83)†	0.72 (0.66–0.79)†
GLP-1a (370)	0.54 (0.44–0.67)†	0.51 (0.41–0.63)†
SGLT-2i (462)	0.48 (0.39–0.58)†	0.44 (0.36–0.54)†

HR, hazard ratio; sHR, subdistribution hazard ratio; CI, confidence interval; TZD, thiazolidinediones; DPP-4i, dipeptidyl-peptidase-4 inhibitors; GLP-1a, glucagon-like peptide-1 agonists; SGLT-2i, sodium-glucose cotransporter-2 inhibitors; PS, propensity score; Table represents time-to-first-dispensation of specific antidiabetic drugs with dementia status as primary exposure; Only subjects without history of specific antidiabetic drug use prior to index date were considered in the analyses (naïve subjects); Attained-age was used as time-scale; Death was considered competing event; Whole-cohort analyses were adjusted for dementia status, index year, sex, diabetes duration and type, Charlson comorbidity index, renal failure, cardiovascular, antithrombotic, antipsychotic, antidepressant, hypnotic/sedative and anxiolytic drugs, education, income group, usage of other antidiabetic drugs and index year; Other antidiabetic medication was considered at the time or prior to index date; Non-diabetic medication use was determined in the interval index date and three-years prior; PS-matched models included one predictor - dementia status; Matching variables included variables used in the whole-cohort analyses, with addition of age; ^*^p < 0.05; ^ †^*p* < 0.001.

**Table 4:** Probability antidiabetic drug dispensation after index date in patients who used metformin in the one-year period prior to index date

	Dementia versus Dementia-free (Whole-cohort analysis)	
	Cox regression Adjusted HR (95% CI)	Competing risk regression Adjusted sHR (95% CI)
Insulin (9,186 new dispensations)	1.33 (1.24–1.42)^ †^	1.24 (1.15–1.33)^ †^
Sulfonylureas (2,881)	0.87 (0.75–1.00)	0.82 (0.71–0.96)^*^
TZD (253)	0.67 (0.39–1.13)	0.64 (0.38–1.06)
DPP-4i (6,999)	0.84 (0.77–0.92)^ †^	0.78 (0.72–0.86)^ †^
GLP-1a (1,539)	0.53 (0.43–0.67)^ †^	0.49 (0.40–0.61)^ †^
SGLT-2i (2,117)	0.50 (0.41–0.61)^ †^	0.47 (0.38–0.57)^ †^
	Dementia versus Dementia-free (PS-matched analysis)	
	Cox regression Crude HR (95% CI)	Competing risk regression Crude sHR (95% CI)
Insulin (1,821 new dispensations)	1.31 (1.19–1.44)^ †^	1.19 (1.08–1.31)^ †^
Sulfonylureas (455)	0.79 (0.66–0.95)^*^	0.74 (0.61–0.89)^*^
TZD (34)	0.92 (0.47–1.81)	0.85 (0.43–1.68)
DPP-4i (1,291)	0.76 (0.68–0.85)^ †^	0.71 (0.64–0.80)^ †^
GLP-1a (263)	0.49 (0.38–0.63)^ †^	0.45 (0.34–0.58)^ †^
SGLT-2i (333)	0.50 (0.40–0.63)^ †^	0.46 (0.37–0.58)^ †^

HR, hazard ratio; sHR, subdistribution hazard ratio; CI, confidence interval; TZD, thiazolidinediones; DPP-4i, dipeptidyl-peptidase-4 inhibitors; GLP-1a, glucagon-like peptide-1 agonists; SGLT-2i, sodium-glucose cotransporter-2 inhibitors; PS, propensity score; Table represents time-to-first-dispensation of specific antidiabetic drugs with dementia status as primary exposure; Only subjects who were users of metformin at the time of or one-year prior to index date and had no history of specific antidiabetic drug use at the time of or prior to index date were considered in the analyses (add-on therapy); Attained-age was used as time-scale; Death was considered competing event; Whole-cohort analyses were adjusted for dementia status, index year, sex, diabetes duration and type, Charlson comorbidity index, renal failure, cardiovascular, antithrombotic, antipsychotic, antidepressant, hypnotic/sedative and anxiolytic drugs, education, income group and other antidiabetic drugs (apart from metformin); Other antidiabetic medication was considered at the time or prior to index date; Non-diabetic medication use was determined in the interval index date and three-years prior; PS-matched models included one predictor - dementia status; Matching variables included variables used in the whole-cohort analyses, with addition of age; ^*^p < 0.05; ^ †^*p* < 0.001.

**Fig. 1.**
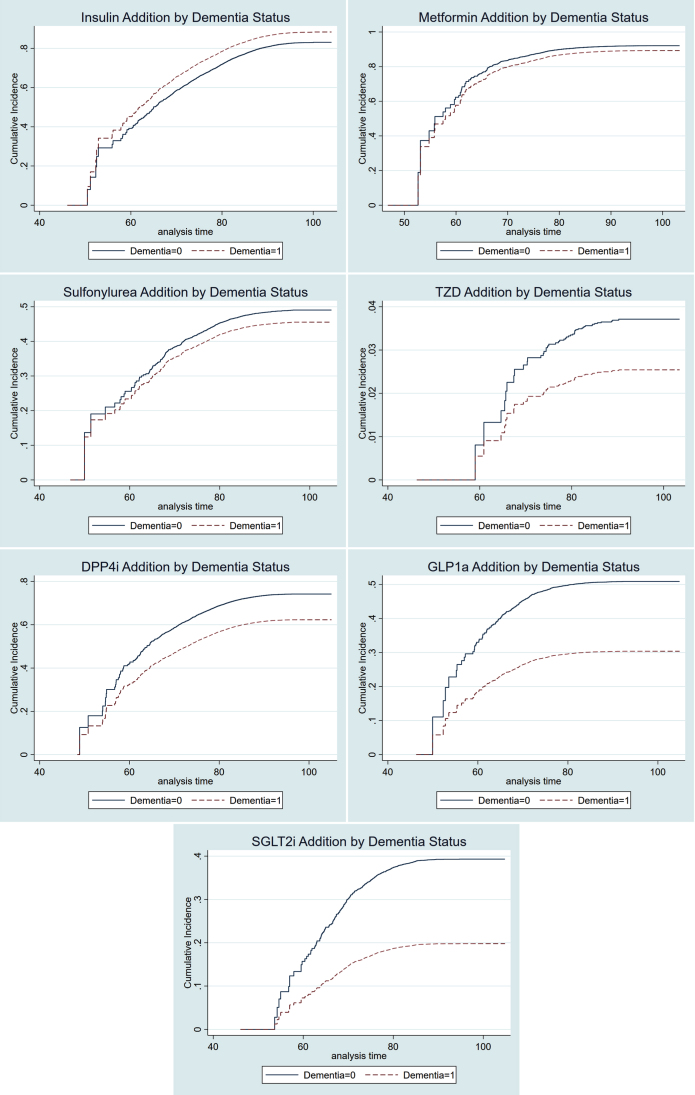
Cumulative incidence functions of antidiabetic drug dispensation in naïve users by dementia status. TZD, thiazolidinediones; DPP-4i, dipeptidyl-peptidase-4 inhibitors; GLP-1a, glucagon-like peptide-1 agonists; SGLT-2i, sodium-glucose cotransporter-2 inhibitors; Curves are based on PS-matched competing risk analyses.

In competing risk models, dementia patients were more likely to be prescribed insulin after index date, in the adjusted whole cohort analyses (sHR 1.22, 95% CI [1.15–1.29]), the PS-matched cohort of all insulin naïve subjects (sHR 1.21, 95% CI [1.11–1.31]), as well as in the PS-matched cohort of metformin users (sHR 1.19, [1.08–1.31]). Moreover, the dementia patients were less likely to be prescribed DPP-4i (sHR 0.72 [0.66–0.79]), GLP-1a (sHR 0.51 [0.41–0.63]), and SGLT-2i (sHR 0.44 [0.36–0.54]) in the PS-matched cohort. Similar probabilities were observed in the whole cohort and in metformin users (Tables 3 and 4). The cumulative incidence functions represent the probability that antidiabetic drug dispensation occurred by certain time and visualize the associations represented by the competing risk estimates in all naïve subjects (matched cohort, Fig. 1).

Analyses stratified on index year showed relatively consistently higher dispensation of insulin in dementia versus non-dementia subjects (Supplementary Figure 4). Conversely, the trend in probability of DPP-4i, GLP-1a, and SGLT-2i dispensation was lower in dementia patients, with a higher degree of uncertainty due to lower number of dispensation events.

## DISCUSSION

To our knowledge, this is the first study to compare the antidiabetic drug prescription patterns in practically all major dementia types during more than a decade of observation. In this large national registry-based cohort, we determined that long-term pharmacological treatment of diabetes differs in patients with dementia in comparison to dementia-free subjects. The drug dispensation pattern we observed indicates a more conservative diabetes management with more extensive insulin utilization and lower probability of receiving DPP-4i, GLP-1a or SGLT-2i when diagnosed with dementia.

The whole cohort experienced frequent insulin dispensation; however, patients with dementia had significantly higher rate of insulin usage and this was apparent even in the yearly-stratified analyses. Older patients with type 2 diabetes receive insulin more frequently [29], and it is likely this trend is extended to patients with dementia. However, the necessity of insulin treatment could function as a proxy for more cardiovascular burden and generally less successful management of hyperglycemia, subsequently leading to higher risk of dementia (reverse causality) [30]. On the other hand, the explanation could lie in simplifying the prescription regimen; Swedish clinicians might be replacing combination treatment (reflected in the declining proportions of metformin and sulfonylureas) with one potent antidiabetic drug. Interestingly, two US studies found a decrease in number of diabetes medications after dementia diagnosis [31, 32], while Weiner and colleagues reported higher use of insulin in patients with poorer health and longer diabetes duration [33].

However, this is unlikely to explain the 21% and 19% higher likelihood of new insulin prescription in naïve subjects and metformin users, respectively. Conceptually, insulin-associated weight gain could be the driving factor, as modest overweight might be protective in this frail patient group [34, 35]. Additionally, if needed, the Swedish patients with dementia and diabetes are provided with nursing assistance regarding insulin injections, therefore the possibility of inappropriate care due to cognitive decline should be decreased. On the other hand, this arrangement puts considerable strain on health care, with unclear frequency of unrecognized hypoglycemia due to decreased sensibility of the patient to its initial symptoms [36].

With such a large proportion of Swedish dementia patients being treated with insulin, we believe periodical continuous glucose monitoring are important especially in patients with advanced dementia or living alone, as cognitive decline increases the probability of hypoglycemia and vice versa [5, 6].

The inverse relationship in dispensation rates suggests a link between the increase in insulin and decrease in metformin and sulfonylurea, independently of dementia or renal status. Importantly, we also observed 26% lower likelihood of sulfonylurea dispensation as add-on therapy to metformin. Lu and colleagues reported corroborating lower utilization of both sulfonylurea and metformin in US dementia patients; however, the authors excluded patients with end-stage renal disease [32]. Chronic renal failure is one of the main contraindications for metformin use, but the minimum estimated glomerular filtration rate for metformin therapy has been revised to at least 30 ml/min/1.73 m^2^ [37]. However, the end of follow-up in our study was in 2018, therefore we could not have caught this revision and it is likely that metformin dispensation has since increased. Interestingly, metformin has previously been associated with cognitive benefit, and was superior in this regard to sulfonylurea and insulin in the recent studies [13, 38].

Decreasing dispensations of sulfonylureas and lower probability of add-on to metformin likely reflect sulfonylureas’ higher propensity towards hypoglycemia, and shorter-acting agents are preferred in the elderly [4]. Overall, sulfonylurea add-on therapy might constitute too big of a risk, and deintensification should be considered especially in advanced ages [39].

The peak of TZD use occurred in the earliest years of observation (years 2005-2007) which reflects the time period when the findings on TZD’s unfavorable cardiovascular profile became known [40]. Afterwards, the prescription rates declined similarly in both dementia and dementia-free cohorts and very few new dispensations occurred. Despite long clinical experience with TZD, their risk-benefit ratio seems too high for broader use in elderly patients with dementia [4, 15].

Importantly, patients with dementia had significantly lower overall dispensation rates of DPP-4i and GLP-1a as well as lower likelihood of being prescribed these medications after dementia diagnosis in all naïve subjects as well as in metformin users. Moreover, the slower initiation of incretin therapy in dementia patients compared to dementia-free controls was observed even in the matched analyses. We believe this finding testifies to a more conservative management of diabetes in Swedish dementia patients, a form of national approach to diabetes care when cognitive dysfunction is present.

Of the two incretin-based therapies, DPP-4i were used more frequently, likely due to their oral form and neutral weight effects [15]. As previously mentioned, the subcutaneous injections of GLP-1a could be a hindrance in dementia patients; however, it is not clear why GLP-1a injections pose a larger barrier than insulin regimen, provided the same assistance. Higher cost of GLP-1a could play a role [4]; however, disposable income did not alter the dispensation probability in our study. On the other hand, decline in weight is commonly noted in patients at risk of dementia [41], and GLP-1a-associated weight loss may be undesirable in patients with fully manifested dementia. Conversely, there is some evidence for decreased risk of dementia associated with incretin therapies [42], as well as alleviation of cerebral brain transport through GLP-1 receptors [43], thus their dispensation could be especially valuable to dementia patients.

SGLT-2i followed a similar pattern of utilization as DPP-4i and GLP-1a, with dementia patients being 56% and 54% less likely to receive new prescription or add-on prescription to metformin of SGLT-2i in the matched analyses. SGLT-2i are on the market since 2011; however, the main breakthrough was established in 2015, with empagliflozin and its unprecedented cardiovascular protection [18]. In general, there is no evidence against SGLT-2i prescription in patients with dementia, but orthostatic hypotension, dehydration, and genital infections are cited as the most common caveats in elderly [4, 15]. However, the combination of blood-pressure reduction, cardiovascular and renal protection could predispose SGLT-2i to reduce the overall pharmacological burden [18]. Definitive data on cognitive functioning are not yet available; however, a small randomized-controlled trial showed SGLT-2i users did not perform worse in cognitive testing after 12 months of treatment [44].

Considering both the pharmacological benefits and lower dispensation of the incretin therapy and SGLT-2i, the question of “missed benefit” in patients with dementia is in order. We propose our findings should be compared with data from other countries, and longitudinal outcomes, such as major hypoglycemic episodes, residual cognitive change, and mortality should be assessed to understand the effectiveness of the Swedish approach.

In conclusion, the Swedish health practitioners seem to prefer insulin management in patients with type 2 diabetes and dementia, while other more recent antidiabetic medications are used less frequently. This is a likely correlate to the care individualization recommended by the current clinical guidelines. To establish how efficient these adjustments were, future studies should assess patient-related outcomes as well as long-term cognitive functioning in relation to specific antidiabetic drug use.

### Strengths and limitations

The major strengths of this study include the large unrestricted dementia cohort, long follow-up in high-quality national registers, as well as the ability to compare the results in dementia-free subjects throughout a wide time interval. Importantly, the range of data in the Drug Register allowed us to include the major antidiabetic drug dispensations across a 14-year time period revolving around index date. Moreover, we used complementary methodological approaches: the whole-cohort and propensity-score matched approach, one providing more generalizable data, and the other achieving higher comparability between subjects. Covariate matching was extensive, which could possibly narrow the covariate imbalance even in the unobserved variables. As we used exact matching on index year to create dementia-dementia-free pairs, the differences in pharmacological care cannot be attributed entirely to evolving diabetes guidelines. Moreover, our results were consistent throughout the analyses of naïve subjects, naïve metformin users as well as yearly-stratified analyses. The national coverage of the supplementary registers is excellent [22, 26–28]. SveDem coverage based on estimated incidence of dementia was estimated to 36% in 2015 [19], however recent data on declining dementia incidence may have underestimated the coverage [45]. We had the opportunity to study non-SveDem dementia cases, but we believe the information on dementia diagnosis originating from other registers is of much lower quality in comparison to SveDem.

A main study limitation is the absence of information reflecting the clinical reasoning for drug prescription, drug side effects or tolerance to specific drugs. It is possible that duration of diabetes is underestimated (lacking information on primary care visits); however, we have no reason to assume differential underestimation between dementia and dementia-free subjects. In addition, we had no biochemical measure of glucose control or estimated glomerular filtration rate, which could provide some insight into the drug dispensation patterns. We were, however, able to adjust and match on the surrogates available –diabetes duration and diagnosis of renal disease. Lastly, the patients’ weight may drive the prescription of specific antidiabetic drugs, unfortunately we had no access to this data. However, our study serves primarily as longitudinal description of drug dispensation within a large, under-researched cohort.

Overall, we believe our study provides substantial information on the long-term drug utilization in a generally understudied population of patients and the results are generalizable to a larger population of dementia patients in Sweden.

## Supplementary Material

Supplementary Material
