# Supplementary material for: Dementia Diagnosis Is Associated with Changes in Antidiabetic Drug Prescription: An Open-Cohort Study of ∼130,000 Swedish Subjects over 14 Years
Source: J Alzheimers Dis. 2020 Aug 18;76(4):1581–94. doi: 10.3233/JAD-200618 (PMC7504989; doi:10.3233/JAD-200618)
Supplement: Supplementary Material [file jad-76-jad200618-s001.pdf]

Supplementary Material

Dementia Diagnosis Is Associated with Changes in Antidiabetic Drug Prescription: An Open-Cohort Study of ~130,000 Swedish Subjects over 14 Years

Supplementary Algorithm 1. Diabetes types according to Patient and Drug Register.

Type 1 diabetes was assigned if:

1. Patient had ICD-10 code E10 in the Patient Register, no other ICD-10 code relevant to diabetes (E11-E14) and had been on insulin-only antidiabetic treatment as recorded in the Drug Register.
2. Patient had ICD-10 code E10 in the Patient Register, as well as other relevant ICD-10 diabetes code (E11-E14), the ICD-10 code E10 was recorded prior to the other diabetes codes, and patient had been on insulin-only antidiabetic treatment as recorded in the Drug Register.

Type 2 diabetes was assigned if:

1. Patient had ICD-10 code E11 in the Patient Register and no other relevant ICD-10 diabetes code was recorded.
2. Patient had ICD-10 code E11 in the Patient Register as well as relevant ICD-10 diabetes codes for type 1 and other/unspecified diabetes, and ICD-10 code E11 was recorded prior to the other diabetes codes.
3. Patient had ICD-10 code E11 in the Patient Register as well as relevant ICD-10 diabetes codes for type 1 diabetes (E10), and ICD-10 code E11 was recorded prior to the ICD-10 code E10, and patient was not on insulin treatment as recorded in the Drug Register.
4. Patient had ICD-10 code E11 in the Patient Register as well as relevant ICD-10 diabetes codes for other/unspecified diabetes (E12-E14), and ICD-10 code E11 was recorded prior to the other diabetes codes.

Other/unspecified diabetes was assigned if:

1. Patient had diagnosis of diabetes but had not fulfilled criteria for type 1 or type 2 diabetes.

Supplementary List 1. ICD-10 codes used as inclusion and exclusion criteria for dementia cases based on Swedish National Patient Register and Swedish Cause of Death Register

Inclusionary codes

a. F00 Dementia in Alzheimer disease
b. F01 Vascular dementia
c. F02 Dementia in other diseases classified elsewhere
d. F03 Unspecified dementia
e. G30 Alzheimer disease
f. Other degenerative diseases of nervous system, not elsewhere classified

Exclusionary codes

a. F05 Delirium, not induced by alcohol and other psychoactive substances
b. F06 Other mental disorders due to brain damage and dysfunction and to physical diseases
c. F07 Personality and behavioral disorders due to brain disease, damage and dysfunction
d. F09 Unspecified organic or symptomatic mental disorder
e. G32 Other degenerative disorders of nervous system in diseases classified elsewhere

Supplementary Figure 1. Study sample selection

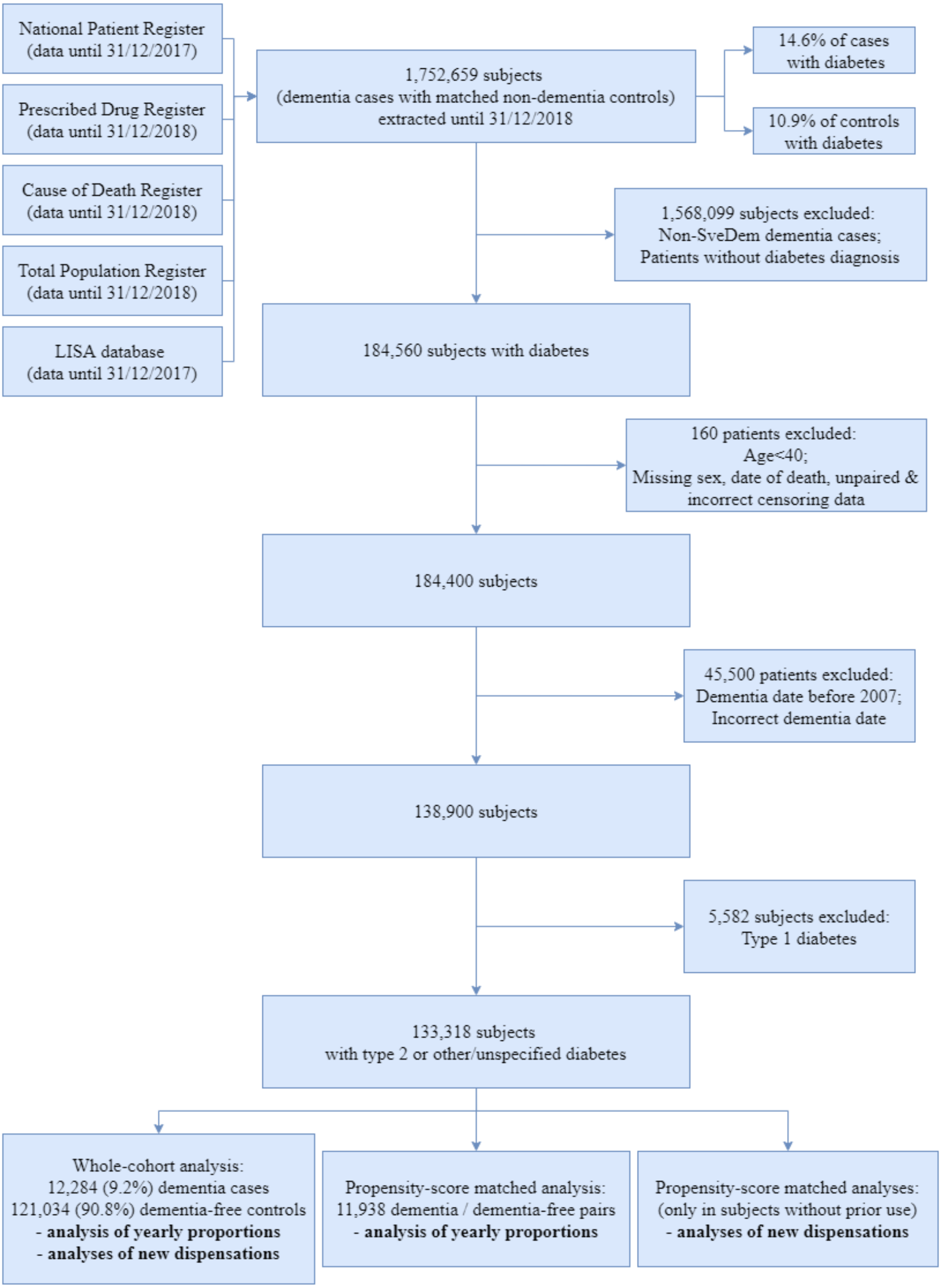

LISA, longitudinal integrated database for health insurance and labour market studies

Supplementary Table 1. Detailed characteristics of the dementia cohort

		**Whole cohort 12,284 subjects**	**PS-matched cohort 11,938 subjects**
Age, years		79.7 (7.2)	79.7 (7.2)
Females		6,302 (51.3%)	6,082 (50.9%)
Cohabitation	Living alone	4,938 (40.5%)	4,800 (40.5%)
	Living with another adult	5,905 (48.5%)	5,742 (48.5%)
	Institutionalized	1,339 (11.0%)	1,299 (11.0%)
Dementia type	Alzheimer's disease	2,714 (22.1%)	2,665 (22.3%)
	Mixed-pathology dementia	2,626 (21.4%)	2,540 (21.3%)
	Vascular dementia	3,405 (27.7%)	3,295 (27.6%)
	Dementia with Lewy bodies	178 (1.4%)	177 (1.5%)
	Frontotemporal dementia	139 (1.1%)	139 (1.2%)
	Parkinson's disease dementia	118 (1.0%)	117 (1.0%)
	Unspecified dementia	2,800 (22.8%)	2,709 (22.7%)
	Other dementia	300 (2.4%)	292 (2.4%)
Mini-Mental State Examination, points		21 (6)	21 (6)
Charlson comorbidity score		2 (2)	2 (2)
Renal disease		825 (6.7%)	807 (6.8%)
Diabetes type	Type 2	7,751 (63.1%)	7,532 (63.1%)
	Other/unspecified	4,533 (36.9%)	4,406 (36.9%)
Diabetes duration, years		7.5 (6.4)	7.5 (6.4)
Cardiovascular drugs		11,707 (95.3%)	11,387 (95.4%)
Antithrombotic drugs		9,148 (74.5%)	8,911 (74.6%)
Antipsychotics		930 (7.6%)	878 (7.4%)
Antidepressants		4,546 (37.0%)	4,396 (36.8%)
Hypnotics/Sedatives		4,218 (34.3%)	4,082 (34.2%)
Anxiolytics		2,917 (23.7%)	2,808 (23.5%)
Education	<9 years compulsory	5,258 (43.9%)	5,247 (44.0%)
	9 years compulsory	896 (7.5%)	892 (7.5%)
	2 years upper secondary	3,092 (25.8%)	3,083 (25.8%)
	3 years upper secondary	1,081 (9.0%)	1,074 (9.0%)
	<3 years college	688 (5.7%)	684 (5.7%)
	3 years college	887 (7.4%)	879 (7.4%)
	Research education	80 (0.7%)	79 (0.7%)
Income category	Low	3,737 (30.4%)	3,541 (29.7%)
	Middle	4,222 (34.4%)	4,118 (34.5%)
	High	4,317 (35.2%)	4,279 (35.8%)
Mortality		6,514 (53.0%)	6,320 (52.9%)

Supplementary Figure 2. Antidiabetic drug proportions in yearly intervals relative to index date (or dementia diagnosis) – whole cohort

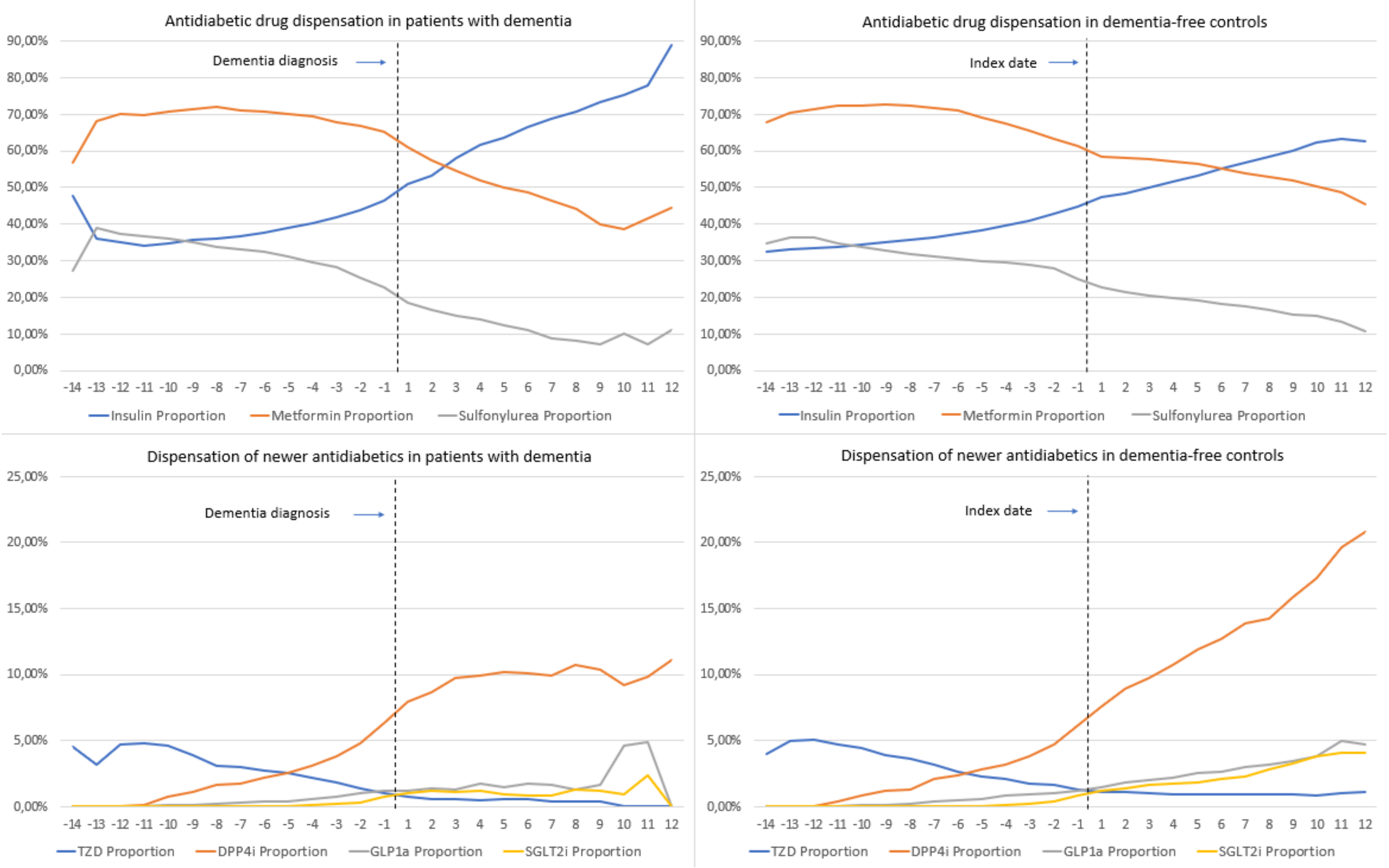

TZD, thiazolidinediones; DPP4i, dipeptidyl-peptidase-4 inhibitors; GLP1a, glucagon-like peptide-1 agonists; SGLT2i, sodium-glucose co-transporter-2 inhibitors; X axis represents yearly intervals relative to index date or dementia date; Proportions were counted as number of specific antidiabetic drug users out of all antidiabetic drugs dispensed in that yearly interval.

Supplementary Figure 3. Antidiabetic drug proportions in yearly intervals relative to index date (or dementia diagnosis) – propensity-score matched cohorts

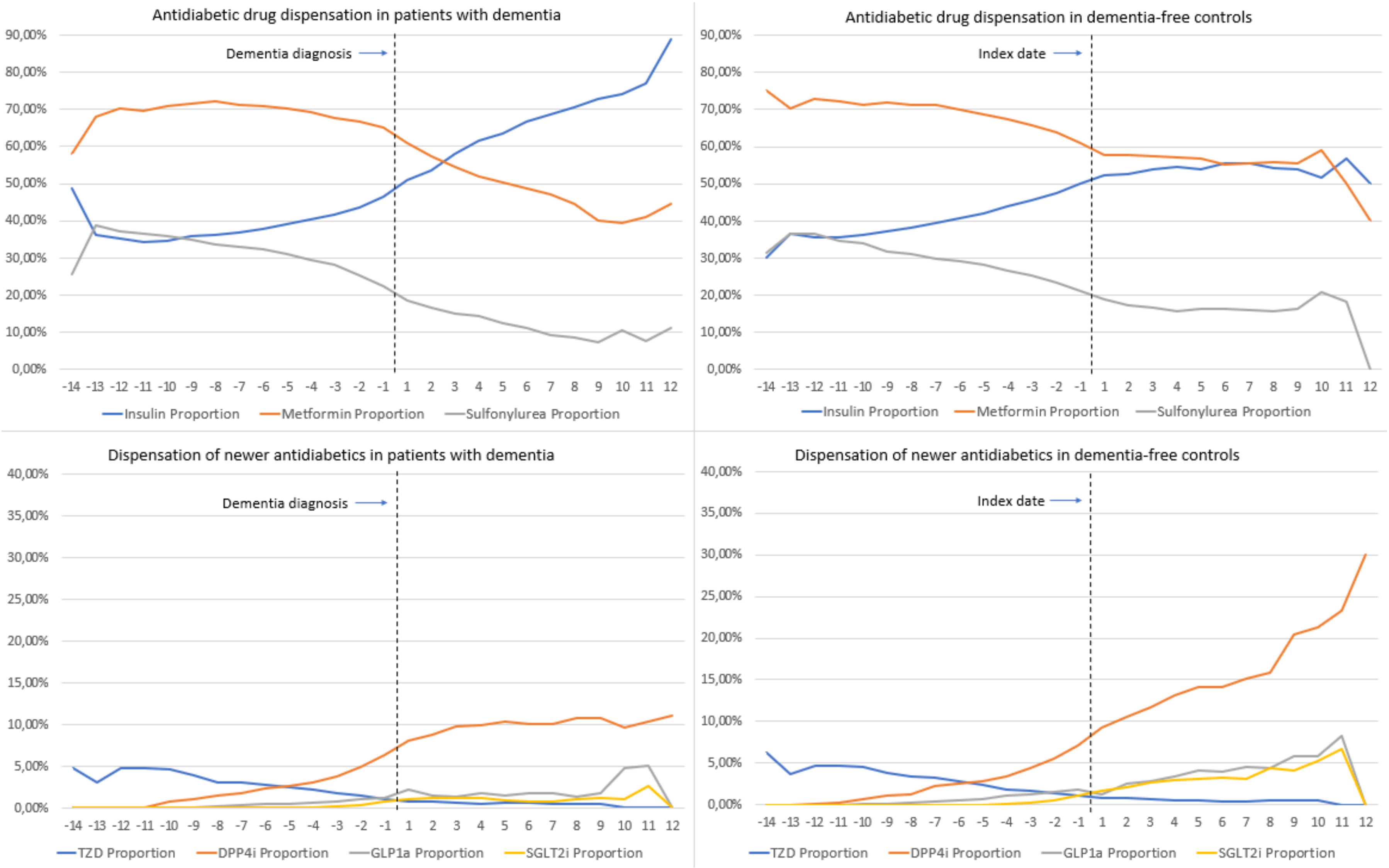

TZD, thiazolidinediones; DPP4i, dipeptidyl-peptidase-4 inhibitors; GLP1a, glucagon-like peptide-1 agonists; SGLT2i, sodium-glucose co-transporter-2 inhibitors; X axis represents yearly intervals relative to index date or dementia date; Proportions were counted as number of specific antidiabetic drug users out of all antidiabetic drugs dispensed in that yearly interval;

Supplementary Figure 4. Probability of antidiabetic drug dispensation after index date in dementia versus non-dementia subjects stratified by year of index date

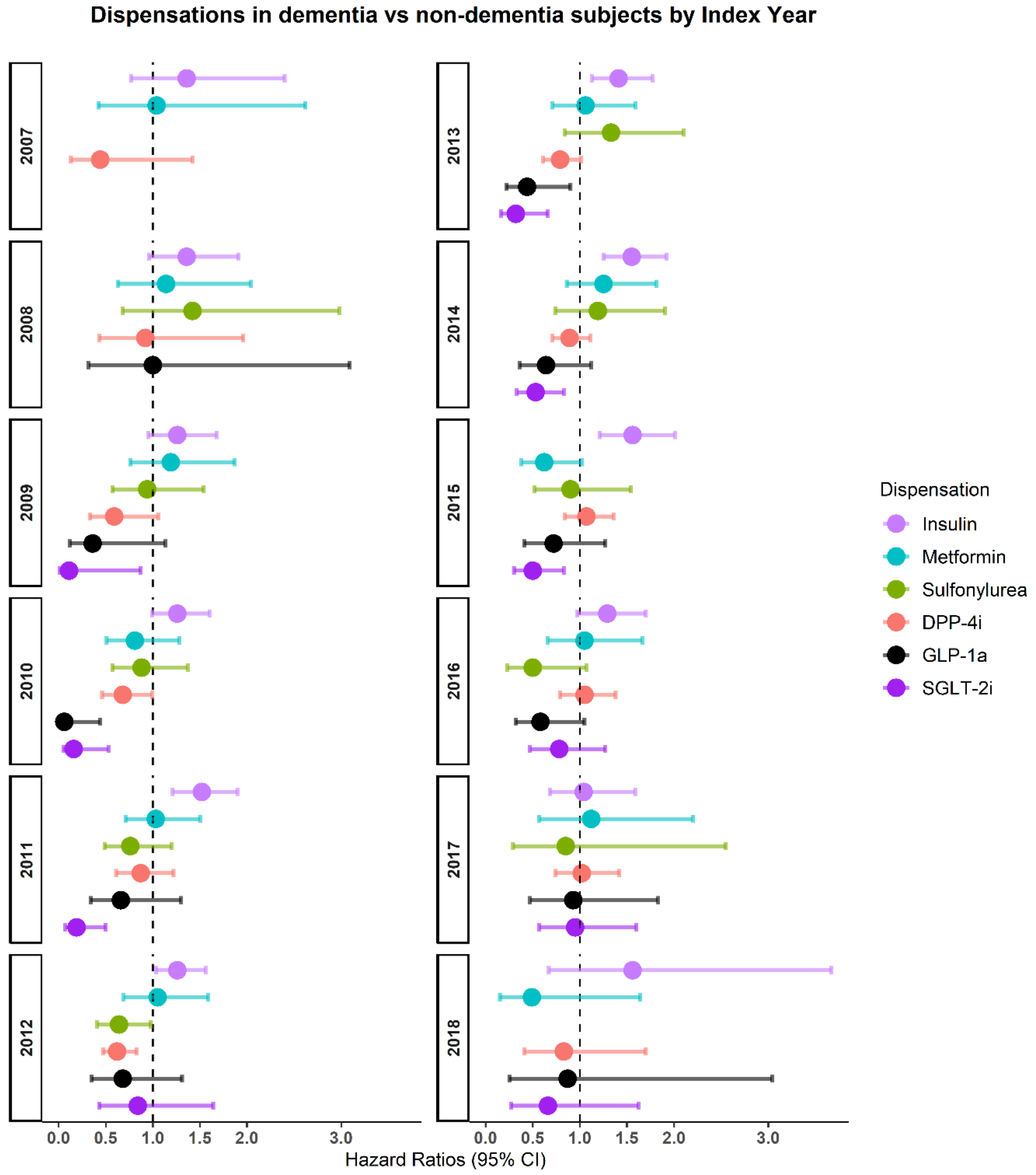

Based on competing-risk regression models in PS-matched cohorts of naïve users; Among patients with index dates in 2007, 2008 and 2018, too few dispensations occurred after index date for some medications; Index Year – year of dementia diagnosis, or year of index date for dementia-free subjects; DPP-4i, dipeptidyl-peptidase-4 inhibitors; GLP-1a, glucagon-like peptide-1 agonists; SGLT-2i, sodium-glucose cotransporter-2 inhibitors

Supplementary Table 2. Balance in the propensity-score matched insulin-naïve subjects

		Diabetes – Insulin-naïve subjects			
		Dementia 7,284 (50.0%)	Dementia-free 7,284 (50.0%)	p	SMD
Age, years		80.0 (7.0)	79.8 (7.3)	0.12	0.03
Female		3,862 (53.0%)	3,960 (54.4%)	0.10	0.03
Diabetes Type	Type 2	4,956 (68.0%)	5,078 (69.7%)	0.20	0.02
	Other/Unspecified	2,328 (32.0%)	2,206 (30.3%)		
Diabetes duration, years		6.1 (6.0)	6.2 (6.1)	<0.001	-0.08
Charlson comorbidity index		2 (2)	2 (3)	<0.001	0.09
Renal disease		304 (4.2%)	288 (4.0%)	0.50	0.01
Cardiovascular drugs		6,883 (94.5%)	6,891 (94.6%)	0.77	-0.01
Antithrombotic drugs		5,239 (71.9%)	5,288 (72.6%)	0.37	-0.02
Antipsychotics		512 (7.0%)	400 (5.5%)	<0.001	0.06
Antidepressants		2,648 (36.4%)	2,774 (38.1%)	0.03	-0.04
Hypnotics/Sedatives		2,480 (34.0%)	2,481 (34.1%)	0.99	-0.01
Anxiolytics		1,700 (23.3%)	1,760 (24.2%)	0.24	-0.02
Education	<9 years compulsory	3,131 (43.0%)	3,083 (42.3%)	0.17	-0.02
	9 years compulsory	546 (7.5%)	501 (6.9%)		
	2 years upper secondary	1,906 (26.2%)	1,964 (27.0%)		
	3 years upper secondary	665 (9.1%)	619 (8.5%)		
	<3 years college	417 (5.7%)	475 (6.5%)		
	3 years college	569 (7.8%)	592 (8.1%)		
	Research education	50 (0.7%)	50 (0.7%)		
Income Category	Low	2,131 (29.3%)	2,048 (28.1%)	0.20	-0.03
	Middle	2,464 (33.8%)	2,456 (33.7%)		
	High	2,689 (36.9%)	2,780 (38.2%)		
Metformin		4,921 (67.6%)	5,091 (69.9%)	0.002	-0.05
Sulfonylurea		2,096 (28.8%)	1,959 (26.9%)	0.011	0.04
Thiazolidinediones		201 (2.8%)	190 (2.6%)	0.57	0.01
GLP-1a		30 (0.4%)	17 (0.2%)	0.058	0.03
DPP-4i		414 (5.7%)	414 (5.7%)	1.00	0.00
SGLT-2i		42 (0.6%)	40 (0.5%)	0.83	0.01

SMD, standardized mean differences in PS-matched dementia cases versus controls; Age is described as mean (SD); Diabetes duration and Charlson comorbidity index are described as median (IQR); Other variables are described as number of patients (%); Age, diabetes duration, Charlson comorbidity index, renal failure, education and income category are described at the time of index date; Antidiabetic medication use was determined at the time or before index date; Other medication use was determined in the interval index date and three-years prior; Dementia – dementia-free pairs were exactly matched on index year; Table describes the subpopulation which did not have insulin prescription prior or at index date - insulin naïve subjects. This subpopulation was used to study the probability of insulin addition after index date.

Supplementary Table 3. Balance in the propensity-score matched metformin-naïve subjects

		Diabetes – Metformin-naïve subjects			
		Dementia 3,578 (50.0%)	Dementia-free 3,578 (50.0%)	p	SMD
Age, years		81.5 (7.1)	81.5 (7.3)	0.91	0.01
Female		1,897 (53.0%)	1,884 (52.7%)	0.76	0.01
Diabetes Type	Type 2	2,326 (65.0%)	2,320 (64.8%)	0.68	-0.01
	Other/Unspecified	1,252 (35.0%)	1,258 (35.2%)		
Diabetes duration, years		6.3 (6.9)	6.6 (7)	<0.001	-0.10
Charlson comorbidity index		2 (2)	2 (2)	<0.001	0.06
Renal disease		322 (9.0%)	313 (8.7%)	0.71	0.01
Cardiovascular drugs		3,343 (93.4%)	3,366 (94.1%)	0.26	-0.03
Antithrombotic drugs		2,676 (74.8%)	2,692 (75.2%)	0.66	-0.01
Antipsychotics		297 (8.3%)	240 (6.7%)	0.01	0.06
Antidepressants		1,301 (36.4%)	1,297 (36.2%)	0.92	0.01
Hypnotics/Sedatives		1,352 (37.8%)	1,287 (36.0%)	0.11	0.04
Anxiolytics		980 (27.4%)	972 (27.2%)	0.83	0.01
Education	<9 years compulsory	1,623 (45.4%)	1,550 (43.3%)	0.03	-0.06
	9 years compulsory	267 (7.5%)	213 (6.0%)		
	2 years upper secondary	900 (25.2%)	943 (26.4%)		
	3 years upper secondary	303 (8.5%)	336 (9.4%)		
	<3 years college	198 (5.5%)	238 (6.7%)		
	3 years college	266 (7.4%)	276 (7.7%)		
	Research education	21 (0.6%)	22 (0.6%)		
Income Category	Low	1,077 (30.1%)	957 (26.7%)	0.005	-0.07
	Middle	1,238 (34.6%)	1,265 (35.4%)		
	High	1,263 (35.3%)	1,356 (37.9%)		
Insulin		1,223 (34.2%)	1,219 (34.1%)	0.92	0.01
Sulfonylurea		984 (27.5%)	910 (25.4%)	0.047	0.05
Thiazolidinediones		63 (1.8%)	55 (1.5%)	0.46	0.02
GLP-1a		8 (0.2%)	12 (0.3%)	0.37	-0.02
DPP-4i		99 (2.8%)	118 (3.3%)	0.19	-0.03
SGLT-2i		3 (0.1%)	3 (0.1%)	1.00	0.00

SMD, standardized mean differences in PS-matched dementia cases versus controls; Age is described as mean (SD); Diabetes duration and Charlson comorbidity index are described as median (IQR); Other variables are described as number of patients (%); Age, diabetes duration, Charlson comorbidity index, renal failure, education and income category are described at the time of index date; Antidiabetic medication use was determined at the time or before index date; Other medication use was determined in the interval index date and three-years prior; Dementia – dementia-free pairs were exactly matched on index year; Table describes the subpopulation which did not have metformin prescription prior or at index date - metformin naïve subjects. This subpopulation was used to study the probability of metformin addition after index date.

Supplementary Table 4. Balance in the propensity-score matched sulfonylurea-naïve subjects

		Diabetes – Sulfonylurea-naïve subjects			
		Dementia<br>7,892 (50.0%)	Dementia-free<br>7,892 (50.0%)	p	SMD
Age, years		79.5 (7.2)	79.4 (7.5)	0.23	0.02
Female		4,083 (51.7%)	4,032 (51.1%)	0.42	-0.01
Diabetes Type	Type 2	4,876 (61.8%)	5,006 (63.4%)	0.10	0.03
	Other/Unspecified	3,016 (38.2%)	2,886 (36.6%)		
Diabetes duration, years		6.5 (6.8)	6.8 (6.7)	<0.001	-0.06
Charlson comorbidity index		2 (2)	2 (2)	<0.001	0.06
Renal disease		496 (6.3%)	510 (6.5%)	0.65	-0.01
Cardiovascular drugs		7,487 (94.9%)	7,508 (95.1%)	0.44	-0.01
Antithrombotic drugs		5,840 (74.0%)	5,841 (74.0%)	0.99	-0.01
Antipsychotics		605 (7.7%)	492 (6.2%)	<0.001	0.05
Antidepressants		2,986 (37.8%)	3,104 (39.3%)	0.054	-0.03
Hypnotics/Sedatives		2,726 (34.5%)	2,753 (34.9%)	0.65	-0.01
Anxiolytics		1,881 (23.8%)	1,888 (23.9%)	0.90	-0.01
Education	<9 years compulsory	3,442 (43.6%)	3,389 (42.9%)	0.088	-0.02
	9 years compulsory	597 (7.6%)	524 (6.6%)		
	2 years upper secondary	2,041 (25.9%)	2,140 (27.1%)		
	3 years upper secondary	710 (9.0%)	687 (8.7%)		
	<3 years college	448 (5.7%)	501 (6.3%)		
	3 years college	603 (7.6%)	604 (7.7%)		
	Research education	51 (0.6%)	47 (0.6%)		
Income Category	Low	2,309 (29.3%)	2,250 (28.5%)	0.09	-0.03
	Middle	2,725 (34.5%)	2,651 (33.6%)		
	High	2,858 (36.2%)	2,991 (37.9%)		
Insulin		2,712 (34.4%)	2,613 (33.1%)	0.10	0.03
Metformin		5,293 (67.1%)	5,466 (69.3%)	0.003	-0.05
Thiazolidinediones		181 (2.3%)	202 (2.6%)	0.28	-0.02
GLP-1a		91 (1.2%)	96 (1.2%)	0.71	-0.01
DPP-4i		406 (5.1%)	395 (5.0%)	0.69	0.01
SGLT-2i		46 (0.6%)	42 (0.5%)	0.67	0.01

SMD, standardized mean differences in PS-matched dementia cases versus controls; Age is described as mean (SD); Diabetes duration and Charlson comorbidity index are described as median (IQR); Other variables are described as number of patients (%); Age, diabetes duration, Charlson comorbidity index, renal failure, education and income category are described at the time of index date; Antidiabetic medication use was determined at the time or before index date; Other medication use was determined in the interval index date and three-years prior; Dementia – dementia-free pairs were exactly matched on index year; Table describes the subpopulation which did not have sulfonylurea prescription prior or at index date - sulfonylurea naïve subjects. This subpopulation was used to study the probability of sulfonylurea addition after index date.

Supplementary Table 5. Balance in the propensity-score matched TZD-naïve subjects

		Diabetes – TZD-naïve subjects			
		Dementia 11,506 (50.0%)	Dementia-free 11,506 (50.0%)	p	SMD
Age, years		79.8 (7.2)	79.7 (7.3)	0.28	0.01
Female		5,626 (48.9%)	5,551 (48.2%)	0.32	0.01
Diabetes Type	Type 2	7,247 (63.0%)	7,364 (64.0%)	0.27	0.02
Diabetes Type	Other/Unspecified	4,259 (37.0%)	4,142 (36.0%)		
Diabetes duration, years		7.4 (6.4)	7.6 (6.4)	<0.001	-0.07
Charlson comorbidity index		2 (2)	2 (2)	<0.001	0.06
Renal disease		757 (6.6%)	754 (6.6%)	0.94	0.01
Cardiovascular drugs		10,961 (95.3%)	11,001 (95.6%)	0.21	-0.02
Antithrombotic drugs		8,583 (74.6%)	8,616 (74.9%)	0.62	-0.01
Antipsychotics		838 (7.3%)	688 (6.0%)	<0.001	0.05
Antidepressants		4,227 (36.7%)	4,302 (37.4%)	0.31	-0.01
Hypnotics/Sedatives		3,912 (34.0%)	3,759 (32.7%)	0.032	0.03
Anxiolytics		2,683 (23.3%)	2,627 (22.8%)	0.38	0.01
Education	<9 years compulsory	5,061 (44.0%)	4,971 (43.2%)	0.013	-0.02
Education	9 years compulsory	857 (7.4%)	765 (6.6%)		
Education	2 years upper secondary	2,968 (25.8%)	3,046 (26.5%)		
Education	3 years upper secondary	1,032 (9.0%)	1,044 (9.1%)		
Education	<3 years college	660 (5.7%)	767 (6.7%)		
Education	3 years college	851 (7.4%)	848 (7.4%)		
Education	Research education	77 (0.7%)	65 (0.6%)		
Income category	Low	3,421 (29.7%)	3,281 (28.5%)	0.021	-0.04
Income category	Middle	3,965 (34.5%)	3,912 (34.0%)		
Income category	High	4,120 (35.8%)	4,313 (37.5%)		
Insulin		4,417 (38.4%)	4,410 (38.3%)	0.92	0.01
Metformin		7,980 (69.4%)	8,262 (71.8%)	<0.001	-0.05
Sulfonylurea		3,781 (32.9%)	3,734 (32.5%)	0.51	0.01
GLP-1a		148 (1.3%)	153 (1.3%)	0.77	-0.01
DPP-4i		770 (6.7%)	815 (7.1%)	0.24	-0.02
SGLT-2i		80 (0.7%)	67 (0.6%)	0.28	0.01

TZD, thiazolidinediones; SMD, standardized mean differences in PS-matched dementia cases versus controls; Age is described as mean (SD); Diabetes duration and Charlson comorbidity index are described as median (IQR); Other variables are described as number of patients (%); Age, diabetes duration, Charlson comorbidity index, renal failure, education and income category are described at the time of index date; Antidiabetic medication use was determined at the time or before index date; Other medication use was determined in the interval index date and three-years prior; Dementia – dementia-free pairs were exactly matched on index year; Table describes the subpopulation which did not have TZD prescription prior or at index date - TZD naïve subjects. This subpopulation was used to study the probability of TZD addition after index date.

Supplementary Table 6. Balance in the propensity-score matched DPP-4i-naïve subjects

		Diabetes – DPP-4i-naïve subjects			
		Dementia 11,081 (50.0%)	Dementia-free 11,081 (50.0%)	p	SMD
Age, years		79.8 (7.2)	79.8 (7.3)	0.50	0.01
Female		5,694 (51.4%)	5,673 (51.2%)	0.78	-0.01
Diabetes Type	Type 2	6,936 (62.6%)	7,182 (64.8%)	0.002	0.04
	Other/Unspecified	4,145 (37.4%)	3,899 (35.2%)		
Diabetes duration, years		7.3 (6.4)	7.5 (6.3)	<0.001	-0.09
Charlson comorbidity index		2 (2)	2 (2)	<0.001	0.08
Renal disease		712 (6.4%)	677 (6.1%)	0.33	0.01
Cardiovascular drugs		10,544 (95.2%)	10,549 (95.2%)	0.88	-0.01
Antithrombotic drugs		8,249 (74.4%)	8,294 (74.8%)	0.49	-0.01
Antipsychotics		813 (7.3%)	667 (6.0%)	<0.001	0.05
Antidepressants		4,111 (37.1%)	4,145 (37.4%)	0.64	-0.01
Hypnotics/Sedatives		3,805 (34.3%)	3,615 (32.6%)	0.007	0.04
Anxiolytics		2,600 (23.5%)	2,542 (22.9%)	0.36	0.01
Education	<9 years compulsory	4,904 (44.3%)	4,739 (42.8%)	0.003	-0.04
	9 years compulsory	817 (7.4%)	734 (6.6%)		
	2 years upper secondary	2,851 (25.7%)	2,983 (26.9%)		
	3 years upper secondary	1000 (9.0%)	985 (8.9%)		
	<3 years college	636 (5.7%)	714 (6.4%)		
	3 years college	800 (7.2%)	869 (7.8%)		
	Research education	73 (0.7%)	57 (0.5%)		
Income category	Low	3,330 (30.1%)	3,120 (28.2%)	0.002	-0.05
	Middle	3,830 (34.6%)	3,818 (34.5%)		
	High	3,921 (35.4%)	4,143 (37.4%)		
Insulin		4,207 (38.0%)	4,121 (37.2%)	0.23	0.02
Metformin		7,590 (68.5%)	7,948 (71.7%)	<0.001	-0.07
Sulfonylurea		3,580 (32.3%)	3,496 (31.5%)	0.23	0.02
TZD		346 (3.1%)	366 (3.3%)	0.45	-0.01
GLP-1a		99 (0.9%)	121 (1.1%)	0.14	-0.02
SGLT-2i		49 (0.4%)	64 (0.6%)	0.16	-0.02

DPP-4i, dipeptidyl-peptidase-4 inhibitors; SMD, standardized mean differences in PS-matched dementia cases versus controls; Age is described as mean (SD); Diabetes duration and Charlson comorbidity index are described as median (IQR); Other variables are described as number of patients (%); Age, diabetes duration, Charlson comorbidity index, renal failure, education and income category are described at the time of index date; Antidiabetic medication use was determined at the time or before index date; Other medication use was determined in the interval index date and three-years prior; Dementia – dementia-free pairs were exactly matched on index year; Table describes the subpopulation which did not have DPP-4i prescription prior or at index date - DPP-4i naïve subjects. This subpopulation was used to study the probability of DPP-4i addition after index date.

Supplementary Table 7. Balance in the propensity-score matched GLP-1a-naïve subjects

		Diabetes – GLP-1a-naïve subjects			
		Dementia 11,771 (50.0%)	Dementia-free 11,771 (50.0%)	p	SMD
Age, years		79.8 (7.1)	79.7 (7.3)	0.42	0.01
Female		6,015 (51.1%)	6,108 (51.9%)	0.23	0.02
Diabetes Type	Type 2	7,432 (63.1%)	7,613 (64.7%)	0.025	0.03
	Other/Unspecified	4,339 (36.9%)	4,158 (35.3%)		
Diabetes duration, years		7.4 (6.4)	7.6 (6.4)	<0.001	-0.07
Charlson comorbidity index		2 (2)	2 (2)	<0.001	0.07
Renal disease		784 (6.7%)	762 (6.5%)	0.56	0.01
Cardiovascular drugs		11,222 (95.3%)	11,218 (95.3%)	0.90	0.01
Antithrombotic drugs		8,782 (74.6%)	8,814 (74.9%)	0.63	-0.01
Antipsychotics		861 (7.3%)	687 (5.9%)	<0.001	0.06
Antidepressants		4,325 (36.7%)	4,449 (37.8%)	0.10	-0.02
Hypnotics/Sedatives		4,023 (34.2%)	4,042 (34.3%)	0.79	-0.01
Anxiolytics		2,769 (23.5%)	2,746 (23.3%)	0.72	0.01
Education	<9 years compulsory	5,188 (44.1%)	5,074 (43.1%)	0.002	-0.03
	9 years compulsory	882 (7.5%)	800 (6.8%)		
	2 years upper secondary	3,026 (25.7%)	3,084 (26.2%)		
	3 years upper secondary	1,061 (9.0%)	1,072 (9.1%)		
	<3 years college	671 (5.7%)	782 (6.6%)		
	3 years college	864 (7.3%)	906 (7.7%)		
	Research education	79 (0.7%)	53 (0.5%)		
Income category	Low	3,495 (29.7%)	3,322 (28.2%)	0.002	-0.05
	Middle	4,057 (34.5%)	3,973 (33.8%)		
	High	4,219 (35.8%)	4,476 (38.0%)		
Insulin		4,511 (38.3%)	4,471 (38.0%)	0.59	0.01
Metformin		8,190 (69.6%)	8,508 (72.3%)	<0.001	-0.06
Sulfonylurea		3,953 (33.6%)	3,815 (32.4%)	0.06	0.02
TZD		411 (3.5%)	426 (3.6%)	0.60	-0.01
DPP-4i		786 (6.7%)	872 (7.4%)	0.03	-0.03
SGLT-2i		64 (0.5%)	53 (0.5%)	0.31	0.01

GLP-1a, glucagon-like peptide-1 agonists; SMD, standardized mean differences in PS-matched dementia cases versus controls; Age is described as mean (SD); Diabetes duration and Charlson comorbidity index are described as median (IQR); Other variables are described as number of patients (%); Age, diabetes duration, Charlson comorbidity index, renal failure, education and income category are described at the time of index date; Antidiabetic medication use was determined at the time or before index date; Other medication use was determined in the interval index date and three-years prior; Dementia – dementia-free pairs were exactly matched on index year; Table describes the subpopulation which did not have GLP-1a prescription prior or at index date - GLP-1a naïve subjects. This subpopulation was used to study the probability of GLP-1a addition after index date.

Supplementary Table 8. Balance in the propensity-score matched SGLT-2i-naïve subjects

		Diabetes – SGLT-2i-naïve subjects			
		Dementia 11,852 (50.0%)	Dementia-free 11,852 (50.0%)	p	SMD
Age, years		79.8 (7.2)	79.7 (7.3)	0.21	0.02
Female		6,051 (51.1%)	6,053 (51.1%)	0.98	0.01
Diabetes Type	Type 2	7,477 (63.1%)	7,658 (64.6%)	0.039	0.03
	Other/Unspecified	4,375 (36.9%)	4,194 (35.4%)		
Diabetes duration, years		7.4 (6.4)	7.8 (6.4)	<0.001	-0.08
Charlson comorbidity index		2 (2)	2 (2)	<0.001	0.07
Renal disease		806 (6.8%)	796 (6.7%)	0.80	0.01
Cardiovascular drugs		11,302 (95.4%)	11,339 (95.7%)	0.25	-0.01
Antithrombotic drugs		8,844 (74.6%)	8,899 (75.1%)	0.41	-0.01
Antipsychotics		867 (7.3%)	700 (5.9%)	<0.001	0.05
Antidepressants		4,367 (36.8%)	4,479 (37.8%)	0.13	-0.02
Hypnotics/Sedatives		4,057 (34.2%)	3,968 (33.5%)	0.22	0.02
Anxiolytics		2,794 (23.6%)	2,781 (23.5%)	0.84	0.01
Education	<9 years compulsory	5,227 (44.1%)	5,145 (43.4%)	0.08	-0.02
	9 years compulsory	883 (7.5%)	782 (6.6%)		
	2 years upper secondary	3,048 (25.7%)	3,181 (26.8%)		
	3 years upper secondary	1,064 (9.0%)	1,080 (9.1%)		
	<3 years college	677 (5.7%)	721 (6.1%)		
	3 years college	876 (7.4%)	871 (7.3%)		
	Research education	77 (0.6%)	72 (0.6%)		
Income category	Low	3,513 (29.6%)	3,309 (27.9%)	0.004	-0.04
	Middle	4,091 (34.5%)	4,088 (34.5%)		
	High	4,248 (35.8%)	4,455 (37.6%)		
Insulin		4,603 (38.8%)	4,616 (38.9%)	0.86	-0.01
Metformin		8,267 (69.8%)	8,679 (73.2%)	<0.001	-0.08
Sulfonylurea		3,992 (33.7%)	4,003 (33.8%)	0.88	-0.01
TZD		426 (3.6%)	474 (4.0%)	0.10	-0.02
GLP-1a		147 (1.2%)	172 (1.5%)	0.16	-0.02
DPP-4i		817 (6.9%)	890 (7.5%)	0.07	-0.02

SGLT-2i, sodium-glucose cotransporter-2 inhibitors; SMD, standardized mean differences in PS-matched dementia cases versus controls; Age is described as mean (SD); Diabetes duration and Charlson comorbidity index are described as median (IQR); Other variables are described as number of patients (%); Age, diabetes duration, Charlson comorbidity index, renal failure, education and income category are described at the time of index date; Antidiabetic medication use was determined at the time or before index date; Other medication use was determined in the interval index date and three-years prior; Dementia – dementia-free pairs were exactly matched on index year; Table describes the subpopulation which did not have SGLT-2i prescription prior or at index date - SGLT-2i naïve subjects. This subpopulation was used to study the probability of SGLT-2i addition after index date.

Supplementary Table 9. Number of propensity-score matched pairs used for the year-stratified analyses with number of dispensations occurred after index date

	Insulin	Metformin	Sulfonylurea	TZD	DPP-4i	GLP-1a	SGLT-2i
2007	69/48	43/18	59/9	n/a	112/13	112/3	112/1
2008	227/132	177/45	209/29	n/a	342/27	343/12	343/7
2009	342/187	233/76	341/62	n/a	570/48	574/15	547/10
2010	500/274	314/74	515/79	n/a	816/111	825/18	828/22
2011	642/314	377/111	707/76	n/a	1,044/129	1,068/35	1,070/31
2012	751/352	392/88	807/87	n/a	1,205/204	1,248/37	1,254/35
2013	795/309	379/95	833/74	n/a	1,244/239	1,291/36	1,304/41
2014	913/345	387/111	1,007/70	n/a	1,406/302	1,489/51	1,504/81
2015	840/245	352/67	948/53	n/a	1,305/262	1,387/50	1,393/66
2016	893/201	383/72	953/30	n/a	1,243/200	1,382/46	1,404/64
2017	850/86	342/34	940/13	n/a	1,152/142	1,293/33	1,310/57
2018	459/24	184/11	563/2	n/a	650/30	753/10	760/20

First number represents the number of pairs / second number represents number of dispensations; TZD, thiazolidinediones; DPP-4i, dipeptidyl-peptidase-4 inhibitors; GLP-1a, glucagon-like peptide-1 agonists; SGLT-2i, sodium-glucose cotransporter-2 inhibitors; TZD were not analysed due to low number of matched pairs and subsequent dispensations
